# Editorial: XVII Spanish Portuguese Congress on Plant Biology (BP2021) - gene expression and genetic modification of plants

**DOI:** 10.3389/fpls.2023.1242609

**Published:** 2023-07-18

**Authors:** Manuel Rey, Marcos Egea-Cortines

**Affiliations:** ^1^ Departamento de Biología Vegetal y Ciencia del Suelo, Universidade de Vigo, Vigo, Spain; ^2^ Genética Molecular, Instituto de Biotecnología Vegetal, Universidad Politécnica de Cartagena, Cartagena, Spain

**Keywords:** plant genetic modification, plant genome editing, plant trypsin inhibitors, ATP-binding cassette protein E2, homologous recombination (HR), CRISPR drive, translation machinery

The XVII Spanish-Portuguese Congress on Plant Biology (BP2021) took place online from 7 to 9 July 2021 due to COVID-19 restrictions. The congress was jointly organized by the Spanish Society of Plant Biology and the Portuguese Society of Plant Physiology with site in the city of Vigo (Galicia, northwestern Spain). The meeting covered both basic and applies subjects in the field of plant biology in 12 scientific sessions. Related to this coverage, Frontiers in Plant Science invited the participants to send manuscripts covering their findings to a Research Topic devoted to Gene Expression and Genetic Modification of Plants. This field of work is gaining recently much interest in the scientific community in particular in relation to new technologies for genetic improvement as genetic edition and epigenetic modifications are achieved. In this Research Topic, 2 original research papers and other 2 Perspective papers specifically focused to Genome Editing in plants were published.

In the first Perspective paper, Tek and Budak introduce the use of CRISPR drives as a new approach to enhance plant pathogen resistance. Gene drives are based on genes cleaving 20-30 nucleotide-sized recognition sites on chromosomes called homing endonuclease genes (HEGs, reviewed by Burt and Koufopanou, 2004). An allele of a diploid organism will only have a 50% chance in normal heredity of being passed on to an offspring. The use of CRISPR/Cas9-based gene drives increase this probability, theoretically up to 100%, although limitations due to efficiency and resistance may exist (Siddiqui et al., 2021). Frequency of transmitting the active genetic element to the next generation is therefore greater than expected by random segregation of heterozygous alleles and is referred to as ‘super-Mendelian’ (Grunwald et al., 2019). This Perspective envisages that CRISPR drives will allow to develop more efficiently resistant cultivars in a shorter time.

DNA double-strand breaks (DSBs) repair (Schmidt et al., 2019) can take place by nonhomologous end joining (NHEJ) or homology-dependent repair (HDR). Chen et al. in the second Perspective paper discuss the strategies directed to improve the efficiency of gene targeting and homologous recombination-based plant genome engineering. Although non-homologous end joining (NHEJ) is the primary mechanism of genome editing in higher plants, it is unpredictable and often produces undesired results. Homology-directed repair (HDR), which proceeds through homologous recombination (HR), is typically the preferred editing method. The competition between HR and NHEJ in repairing DSBs has been observed in many species, including plants (Manova and Gruszka, 2015; Schmidt et al., 2019). In their perspective, Chen et al. review the potential and challenges of HR for gene editing of plants, expecting that the combination of several strategies may improve the possibilities of altering plant genomes with precision for crop improvement and basic science research.

As plant trypsin inhibitors (TI) have negative effects on the digestive system of herbivores (Ryan, 1990), Sultana et al. briefly review the effects of overexpressing TI genes in several plants. With the goal of using TI synthesis in the leaves of plants as a possible effective strategy to provide resistance against leaf defoliating insects, Sultana et al. engineered Arabidopsis and soybean plants by overexpressing soybean TI genes under the control of the constitutive CaMV 35S promoter or the green tissue-specific *rbcs-SRS4* gene promoter. Their results using *in vitro* enzyme assays and insect bioassays indicate that TI are able to inactivate insect digestive enzymes, with a significant reduction in larval weight and a significant reduction of leaf defoliation compared to non-transgenic plants. This work highlights the potential benefits for crop and environment protection with reduced chemical applications by using inherent defensive proteins in plants.

The ATP-Binding Cassette E (ABCE) proteins are soluble ABC proteins involved in ribosome recycling and translation initiation as it was studied in archaea, fungi, or animals (Navarro-Quiles et al., 2018), but their roles in plants remain unclear. In the last paper of this Research Topic, Navarro-Quiles et al. report their results of a functional analysis of the Arabidopsis *ABCE2* gene. They found that Arabidopsis has two *ABCE* paralogs, of which *ABCE2* seems to conserve the ancestral function. Navarro-Quiles et al. found that ABCE2 physically interacts with components of the translation machinery, and their RNA-seq study showed increased responses to iron and sulfur deficiencies, as well as the upregulation of auxin signaling and primary metabolism genes. These results support a conserved role for ABCE proteins in translation in plants, and the ABCE2 protein seems important for general growth and vascular development in Arabidopsis, maybe due to an indirect effect through auxin metabolism.

As a general conclusion, contributions to this Research Topic present novel insights in understanding basic gene expression mechanisms with potential usefulness for modification of plants for agricultural and environmental challenges.

## Author contributions

MR and ME-C contributed to the writing of this editorial. All authors contributed to the article and approved the submitted version of this editorial.
